# Sea buckthorn berry extract favorably affects bone metabolism-related biomarkers and collagen deposition in cultured rat primary osteoblasts

**DOI:** 10.1515/biol-2025-1288

**Published:** 2026-02-23

**Authors:** Monika Martiniakova, Vladimira Mondockova, Anna Sarocka, Noemi Penzes, Veronika Kovacova, Roman Biro, Natalia Slawinska, Beata Olas, Radoslav Omelka

**Affiliations:** Department of Zoology and Anthropology, Faculty of Natural Sciences and Informatics, Constantine the Philosopher University in Nitra, Nitra, Slovakia; Department of Botany and Genetics, Faculty of Natural Sciences and Informatics, Constantine the Philosopher University in Nitra, Nitra, Slovakia; Department of General Biochemistry, Faculty of Biology and Environmental Protection, University of Lodz, Lodz, Poland

**Keywords:** sea buckthorn, fruit extract, primary osteoblasts, biomarkers, collagen, *in vitro*

## Abstract

This study aimed to analyse the impact of sea buckthorn (SB) berry extract on the function of cultured rat primary osteoblasts, including the production of bone metabolism-related biomarkers and bone matrix formation. Primary osteoblasts best reflect *in vivo* conditions. Osteoblast apoptosis, viability, alkaline phosphatase (ALPL) activity, production of ALPL, osteocalcin (BGLAP), collagen type I alpha 1 (COL1A1), integrin-binding sialoprotein (IBSP), tumor necrosis factor ligand superfamily member 11 (TNFSF11), and calcium/collagen deposition were assessed. The composition of the extract showed that the main phenolic metabolites found were flavonol glycosides (67.1 %). SB berry extract significantly increased the levels of BGLAP (at 0.5 and 1 μg/mL), COL1A1 (at 1–100 μg/mL), IBSP (at 0.1–1 μg/mL), collagen deposition (at 1–10 μg/mL), and decreased TNFSF11 levels (at 0.1 and 0.5 μg/mL). Although higher doses of the extract (50 and 100 μg/mL) reduced osteoblast apoptosis, they also lowered cell viability, IBSP levels, and mineralization. It can be concluded that SB berry extract at concentrations up to 10 μg/mL favorably affected multiple bone metabolism-related biomarkers, indicating that it has encouraging potential for use as a nutraceutical to support bone health due to the unique composition of bioactive metabolites and the known synergistic interactions between them.

## 1 Introduction

Sea buckthorn (SB, *Elaeagnus rhamnoides* (L.) A. Nelson) is a flowering shrub that contains various bioactive metabolites with antioxidant, anti-inflammatory, anti-tumor, and immunomodulatory activities in its parts, including berries, seeds, and leaves. Phenolic metabolites (such as flavonoids and phenolic acids), phytosterols, triterpenoids, carotenoids, amino acids, organic acids, fatty acids, vitamins, and minerals are among the approximately 200 bioactive compounds discovered in this plant [1], 2]. Flavonoids, primarily isorhamnetin, quercetin, and kaempferol, are the most common active metabolites in SB, which are believed to support bone health [3], 4]. Numerous *in vitro* investigations have demonstrated their capacity to suppress oxidative stress, osteoblast apoptosis, and osteoclastogenesis [5], [6], [7]. Furthermore, the osteoprotective effects of quercetin and kaempferol have also been discovered in *in vivo* studies [8], 9]. Considering SB, it has shown significant promise in the treatment of osteoporosis, the most prevalent bone disease, according to *in vivo* research employing suitable animal models of osteoporosis. In ovariectomized (OVX) rats, Yuan et al. [10] reported that SB could improve trabecular bone microarchitecture, elevate levels of bone turnover markers, estrogen, as well as raise bone mineral density (BMD). Alcoholic extract of SB fruits increased BMD and had protective benefits against cartilage degradation and disrupted trabecular bone microarchitecture even in OVX mice [11]. Current studies suggest that the levels of bone formation and mineralization-related biomarkers, including alkaline phosphatase (ALPL), osteocalcin (BGLAP), collagen type I alpha 1 (COL1A1), integrin-binding sialoprotein (IBSP), may be positively affected by the ability of SB to promote osteoblast differentiation *in vitro*. Lee et al. [12] tested isolated bioactive compounds from SB fruit extract in mouse mesenchymal stem cells (MSCs, a line C3H10T1/2) to evaluate the efficiency of promoting osteoblast differentiation. They found that triterpenes increased gene expression of osteogenesis-related biomarkers, suggesting that the extract could favorably influence bone health. Park et al. [11] revealed that SB fruit extract elevated osteoblast differentiation in mouse C3H10T1/2 cells and enhanced osteogenic gene expression. The effect of SB fruit extract on cultured primary osteoblasts has not been evaluated yet, despite the fact that these cells best reflect *in vivo* conditions compared to other osteoblast-like cell lines. Therefore, the objective of this *in vitro* study was to analyse the impact of SB berry extract (a polyphenol-rich fraction from fruits) on osteoblast apoptosis, viability, ALPL activity, production of specific bone metabolism-related biomarkers, and calcium/collagen deposition in rat primary osteoblast cell culture.

## 2 Materials and methods

### 2.1 Primary osteoblast culture

Calvarial rat primary osteoblasts, purchased from InnoProt (Rat Osteoblasts, P10931, Derio, Spain), were cultured in alpha minimum essential medium (alpha-MEM) (Sigma-Aldrich, St. Louis, MO, USA) supplemented with antibiotics (1 % penicillin-streptomycin solution, HyClone, Logan, UT, USA) and 10 % fetal bovine serum (FBS; Sigma-Aldrich). Cells were cultured at 37 °C in a 5 % CO_2_ atmosphere in 75 cm^2^ culture flasks until 80–90 % confluence was reached. Culture medium was changed every 2 days. Subculture was carried out according to Taylor et al. [13]. All experimental analyses were performed during the exponential growth phase after the third passage. Osteoblasts were incubated without (control group) and with SB berry extract, which was obtained from the University of Lodz (Poland). The conducted research was not related to either human or animals use.

### 2.2 SB berry extract

The extract was prepared from freeze-dried SB fruits (800 g) that were subjected to cold extraction with 4 L of 80 % methanol (v/v; 24 h), assisted with ultrasonic treatment (2 × 10 min). The material was further extracted with boiling 80 % methanol (v/v; 4 L; 1 h), under reflux. Both extracts were combined, filtered, and concentrated in a rotary evaporator (Heidolph, Schwabach, Germany) to remove the organic solvent. The residue was applied onto a short LiChroprep 40–63 μm RP-18 column (Millipore Corp., Bedford, MA) and equilibrated with water. After washing the column with water to remove highly polar constituents, bound phenolic compounds were eluted with 50 % methanol (v/v). The obtained eluate was concentrated by a rotary evaporation and subsequently freeze-dried, to yield 10.19 g of dry phenolic extract. Subsequently, the resulting extract was analysed by high-performance liquid chromatography coupled with mass spectrometry (LC-MS) as previously described [14]. The extract was dissolved in culture medium at final concentrations of 0.1, 0.5, 1, 5, 10, 50, and 100 μg/mL.

### 2.3 Osteoblast biomarkers

The cells were cultured in alpha-MEM and treated for 72 h. Osteoblast apoptosis was evaluated using the terminal deoxynucleotidyl transferase dUTP nick-end labeling (TUNEL) assay and deoxyribonucleic acid (DNA) fragmentation was quantified using the HT TiterTACS™ Apoptosis Detection Kit (Trevigen, Gaithersburg, MD, USA). The proportion of DNA-fragmented (apoptotic) cells was calculated as a percentage relative to the total cell number. Cell viability was assessed by the colorimetric MTS assay (CellTiter 96^®^ AQueous One Solution Assay, Promega, Madison, WI, USA) and the results were expressed as a percentage of optical density (OD) relative to the control group. ALPL activity was detected using BCIP^®^/NBT SIGMAFAST™ substrate, which stains cells blue-violet in the presence of ALPL activity. Staining intensity was quantified spectrophotometrically. Concentrations of specific bone metabolism-related proteins, such as ALPL, COL1A1, IBSP, BGLAP, tumor necrosis factor ligand superfamily member 11 (TNFSF11 or RANKL) were determined using enzyme-linked immunosorbent assay (ELISA) kits (cat. no. ER0728, ER0850, ER0777, ER1205-HS, ER1604; FineTest, Wuhan, China). ALPL, COL1A1, IBSP, and BGLAP were quantified in culture medium, RANKL was analysed in cell lysates.

### 2.4 Calcium and collagen deposition

In a mineralization assay, treated and control cells were cultivated in osteogenic medium for 21 days. The osteogenic medium consisted of alpha-MEM supplemented with 10 % FBS, 1 % antibiotics, 5 mM β-glycerophosphate, 50 μg/mL ascorbic acid, and 10 nM dexamethasone. Quantification of calcium deposits was performed using the Alizarin Red S Staining Quantification Assay (cat. no. 8678, ScienCell Research Laboratories, USA). Collagen deposition was calculated from absorbance measurements after culturing osteoblasts in alpha-MEM medium for 21 days and staining the cells with 0.1 % Sirius Red in saturated aqueous picric acid.

### 2.5 Statistical analysis

Statistical analysis was performed using SPSS v. 26.0 software (IBM Corp., Armonk, NY, USA). The results were presented as mean ± standard deviation (SD) of experiments measured as triplicate with three or eight (determination of viability, apoptosis, and ALPL activity) technical replicates. The data obtained were evaluated using one-way analysis of variance (ANOVA) with Games-Howell or Tukey’s post hoc tests. P values lower than 0.05 were considered statistically significant.

## 3 Results

### 3.1 SB berry extract composition

LC-MS analysis of the extract confirmed that the main compounds identified were flavonol glycosides (67.1 % of relative peak area), especially isorhamnetin glycosides (54 %), quercetin glycosides (12.1 %), kaempferol glycosides (1 %), catechins (0.5 %), and unidentified non-polar compounds (2.4 %). Additionally, SB berry extract contained unidentified polar compounds (20.9 %) and triterpenes (9.1 %).

### 3.2 Osteoblast biomarkers

**Figure 1:**
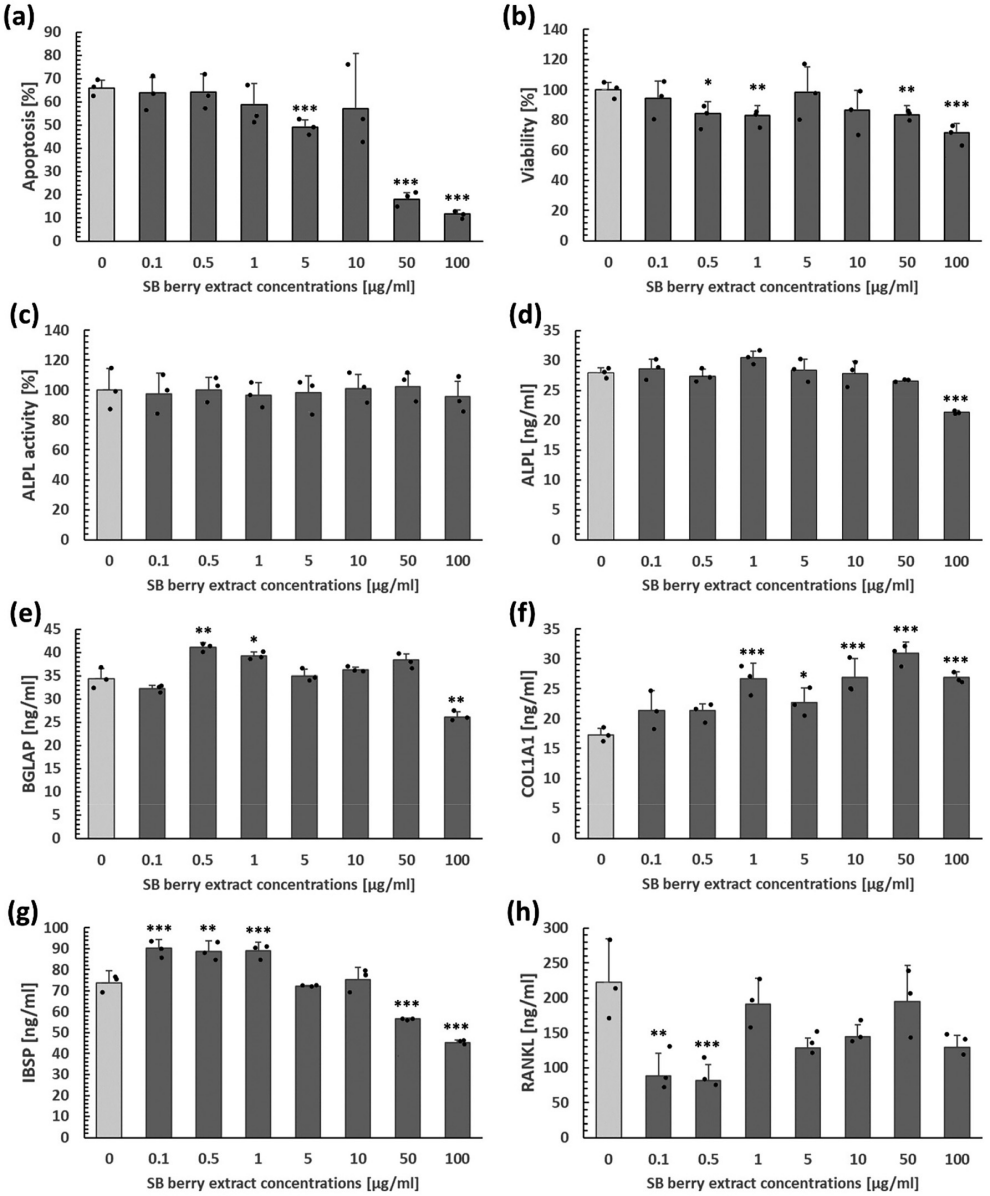
Osteoblast apoptosis (a), viability (b), ALPL activity (c), and the levels of ALPL (d), BGLAP (e), COL1A1 (f), IBSP (g), and RANKL (h) in cultured rat primary osteoblasts after SB berry extract administration at doses of 0–100 μg/ml *(p < 0.05), **(p < 0.01), ***(p < 0.001).

Extract concentrations of 0.1–10 μg/mL did not affect osteoblast apoptosis, but higher concentrations (50 and 100 μg/mL) significantly reduced it. In contrast, only doses of 0.1, 5, and 10 μg/mL reached the level of viability of control cells. Considering the biomarkers of bone formation, no extract concentrations had an effect on ALPL activity. ALPL levels remained unchanged after administration of the extract at doses ranging from 0.1 to 50 μg/mL. Concentrations of 0.5 and 1 μg/mL elevated BGLAP levels. Conversely, the dose of 100 μg/mL significantly reduced the production of both BGLAP and ALPL. These findings demonstrate that SB berry extract has no detrimental effects on bone formation and may potentially improve systemic bone health by increasing BGLAP. Furthermore, the extract increased the concentrations of COL1A1 (at 1–100 μg/mL), IBSP (at 0.1–1 μg/mL), and decreased the level of TNFSF11 at doses of 0.1 and 0.5 μg/mL (Figure 1). The beneficial effect of SB berry extract on COL1A1 and IBSP production indicates a direct impact of its bioactive metabolites on important structural and functional indicators of bone. The downregulation of TNFSF11 at lower extract doses points to the direct involvement of SB in reducing bone resorption.

### 3.3 Calcium and collagen deposition

**Figure 2:**
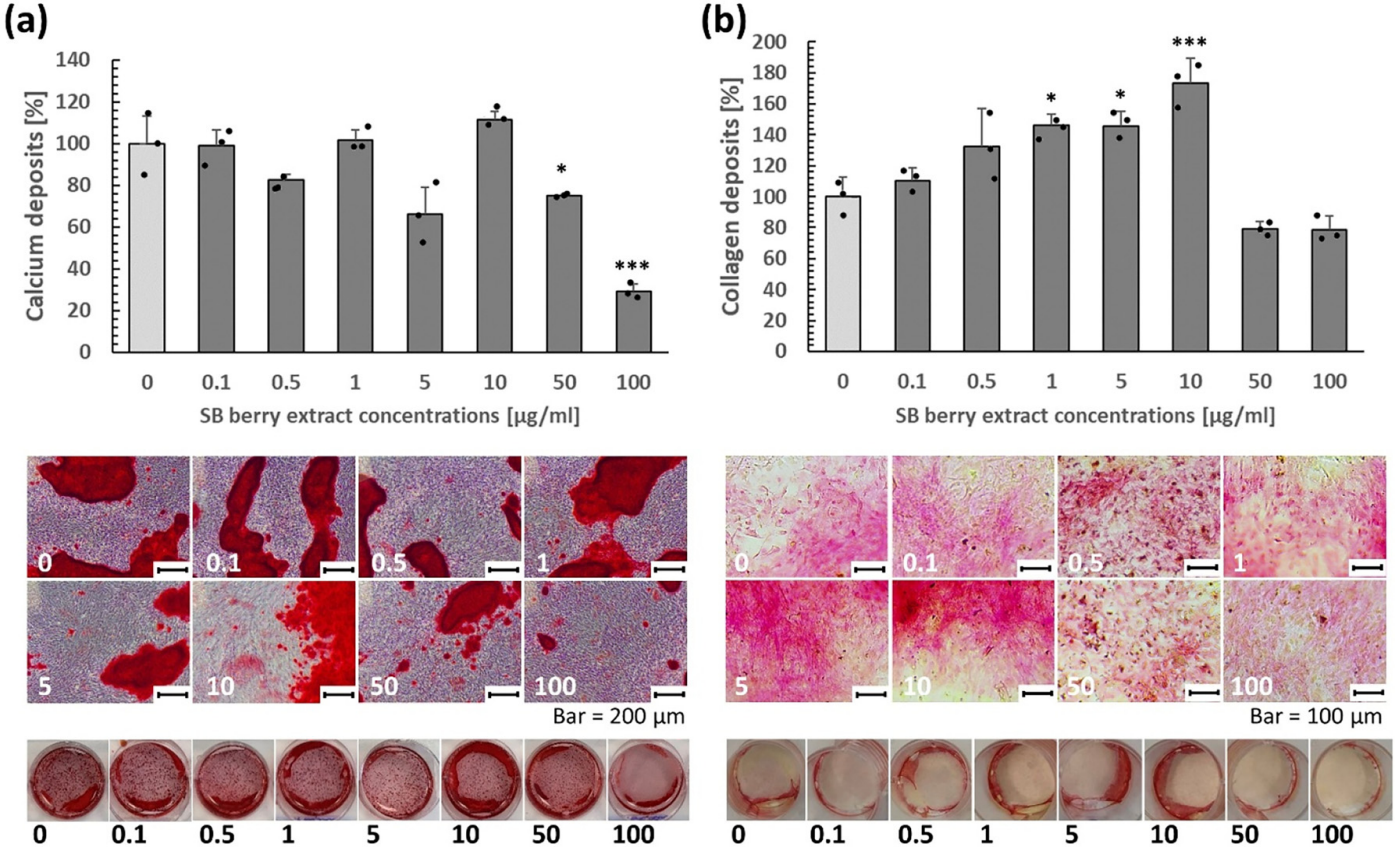
Deposition of calcium (a) and collagen (b) by cultured rat primary osteoblasts after SB berry extract administration at doses of 0–100 μg/ml. Measurements, representative microphotographs, and whole-well images from a 6-well (for calcium deposition) and 12-well (for collagen deposition) plate are presented. *(p < 0.05), ***(p < 0.001).

Mineralization of osteoblasts at SB berry extract concentrations up to 10 μg/mL reached the same level as control cells cultured in mineralization medium. At other concentrations, calcium deposition was lower. However, extract doses of 1–10 μg/mL increased collagen deposition (Figure 2), which (together with the calcium deposition results) indicates a positive effect on bone matrix formation.

## 4 Discussion

Skeletal processes have been studied using various cell lines, including bone marrow MSCs (with the ability to differentiate into osteoblasts), macrophage-like cells (RAW 264.7), and osteoblast-like cells (MC3T3-E1). In our research, primary osteoblasts were used to avoid any cell-specific responses and also to better reflect the mechanisms under *in vivo* conditions. In general, rat primary osteoblasts are widely recognized as an appropriate preclinical model due to their reproducible differentiation and mineralization patterns, which resemble those of human osteoblasts [13], 15]. Recently, 3D culture models or on-a-chip approaches have expanded the possibilities of more accurately mimicking *in vivo* conditions for osteoblasts and other bone cell types and co-cultures [16], 17]. In general, the use of primary osteoblasts offers a powerful platform to assess the effects of different drugs before their implementation in clinical trials. Osteoblast cultures are therefore valuable preclinical tools [18] and frame SB berry extract as a potential therapeutic screening candidate within modern toxicity-testing paradigms.

According to our results, only doses of 0.1, 5, and 10 μg/mL reached the level of viability of control cells. Nevertheless, the production of investigated biomarkers, as well as the deposition of calcium and collagen, were not significantly influenced, although cell viability was reduced at SB berry extract doses of 0.5 and 1 μg/ml. On the other hand, higher viability-reducing doses of the extract (especially 100 μg/mL) negatively affected the production of certain biomarkers and calcium deposition. It is widely recognized that high doses of flavonoids support cytotoxicity and cell death induction by increasing oxidative stress, a mechanism exploited in cancer biology [19]. Among several factors, cytoskeletal proteins and membrane repair mechanisms are crucial for the viability and signaling of bone cells exposed to stress [20], 21]. Significantly reduced osteoblast apoptosis at high concentrations of SB berry extract could indicate the triggering of other forms of cell death, indicating a dose-dependent, complex effect on the balance of bone cell life/death.

Our findings demonstrated that collagen deposition in cultured rat primary osteoblasts and multiple biomarkers related to bone metabolism, such as BGLAP, COL1A1, IBSP, TNFSF11, were favorably affected by SB berry extract at concentrations up to 10 μg/mL. None of the aforementioned biomarkers were significantly reduced below the level of control osteoblasts at the indicated extract concentrations. These are the first results performed on primary osteoblasts.

The composition of SB berry extract indicated that the primary phenolic metabolites found were flavonoids (specifically flavonol glycosides), which is consistent with published research that has discovered such metabolites in the extract [14], [22], [23], [24], [25]. Overall, some flavonoids, such as isorhamnetin and quercetin, quercetin and kaempferol, kaempferol and catechins, have been shown to exhibit synergistic effects in various cell lines [3], 26]. Li et al. [27] reported that isorhamnetin (at 2.5–10 µM), a crucial flavonol in SB berry extract, enhanced the differentiation and mineralization of preosteoblast MC3T3-E1 cells. In RAW 264.7 cells (a precursor for osteoclasts), isorhamnetin suppressed RANKL-induced osteoclastogenesis [28], suggesting its beneficial impact on osteoblastogenesis. Quercetin (at 200 mM) increased the proliferation, differentiation, and mineralization of MC3T3-E1 cells, as well as the activity of osteogenesis-related biomarkers (e.g. ALPL, COL1A1, IBSP, BGLAP). Conversely, it inhibited the proliferation and differentiation of RAW 264.7 cells [29], similar to isorhamnetin. Kaempferol (at 0.1–10 µM) and catechins (at 1 µM and 10 µM) also promoted osteogenic differentiation of bone marrow MSCs as well as MC3T3-E1 osteoblast-like cells through enhanced expression of osteoblast-activated factors (e.g. ALPL, COL1A1), elevated ALPL activity, and calcium deposition [30], [31], [32]. The SB berry extract used in our study also contained triterpenes, similar to other studies [22], 25]. Their ability to support osteoblastogenesis in MSCs has already been demonstrated [12]. In addition, synergistic activity between triterpenoids and flavonoids was detected [33]. Unidentified polar compounds were also identified in the extract, which is consistent with the study by Sławińska et al. [23]. These compounds are thought to interact with pathways that promote osteoblast differentiation and activity [34]. In this regard, it is recognized that musculoskeletal health can be improved by molecular targeting of systemic pathways, such as AhR signaling [35].

Recently, several investigations have suggested that the overall activity of the extract is the result of interactions between their metabolites [36], 37]. Therefore, we suppose that the beneficial impact of SB berry extract on bone health is due to the unique composition of bioactive metabolites, as well as the known synergistic interactions between them. In this context, our study provides insight into the modulation of cellular functions by the action of SB extract in its specific composition, which, together with *in vivo* studies, provides a basis for designing subsequent clinical trials.

Our study has some limitations. Although the SB berry extract was chemically profiled, the main constituents were not tested individually, making it difficult to identify which molecule(s) drive the observed effects and to confirm synergistic interactions. Accordingly, individual metabolites should be tested separately in future studies. Furthermore, the bioavailability of flavonoids is generally low due to interactions with serum proteins. Further studies would also be needed to estimate the amount of unbound bioactive components of the extract available to cells [38]. In addition, primary rat osteoblasts, which are already largely differentiated, were used in our experiment. They offer a limited dynamic range to detect pro-differentiation effects and may add little to the mechanistic case. On the other hand, this type of cells best reflects *in vivo* conditions compared to other osteoblast-like cell lines. Moreover, our study presents initial results of the effect of SB berry extract on primary osteoblasts.

## 5 Conclusions

SB berry extract at concentrations up to 10 μg/mL favorably affected bone metabolism-related biomarkers, including BGLAP, COL1A1, IBSP, TNFSF11, as well as collagen deposition in cultured rat primary osteoblasts due to the unique composition of bioactive compounds, as demonstrated by LC-MS analysis, and possible synergistic effects between multiple metabolites. Consequently, SB berry extract shows encouraging potential for use as a nutraceutical to promote bone health.
